# Anharmonic Assignment of the Water Octamer Spectrum in the OH Stretch Region

**DOI:** 10.1021/acs.jpca.3c02902

**Published:** 2023-07-21

**Authors:** Davide Barbiero, Gianluca Bertaina, Michele Ceotto, Riccardo Conte

**Affiliations:** † Dipartimento di Chimica, Università degli Studi di Milano, via Golgi 19, 20133 Milano, Italy; ‡ Istituto Nazionale di Ricerca Metrologica, Strada delle Cacce 91, I-10135 Torino, Italy

## Abstract

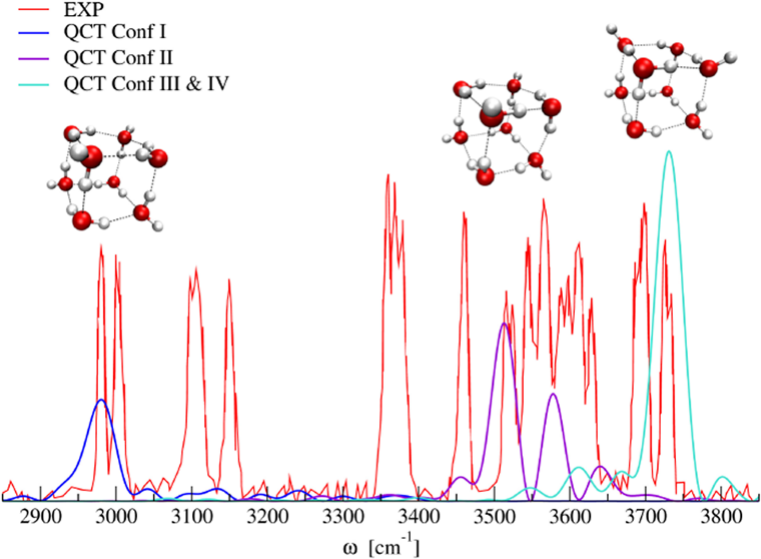

We interface the quasi-classical trajectory approach with an *ab initio* potential energy surface for water to assign the vibrational spectroscopical features of the OH stretch region of the water octamer cluster, which is considered to be a precursor of ice. An attempt by Li et al. to assign their recent reference experiment involved lower-level calculations based on an *ad hoc* scaled harmonic approach. Differently from the conclusions of this previous assignment, which invoked the contribution of 5 conformers and a solvated form of the water heptamer in the spectrum, we find out that the spectroscopic features can be related to the 4 conformers of the octamer lying lower in energy.

## Introduction

Water has always attracted a great deal of attention due to its unique properties like high surface tension and boiling point, a negative slope in the phase diagram for the solid–liquid equilibrium and many others, which altogether make it an essential component for life. It is well known that hydrogen bonding plays a major role in all of them. For this reason, extensive efforts have been devoted to the spectroscopic characterization of neutral and protonated water clusters since the OH stretch modes of water can provide detailed information at the microscopic level on the hydrogen bond structure.^1,2^ Remarkably, previous experimental and theoretical work revealed that the water trimer, tetramer, and pentamer all have cyclic global minimum structures with all oxygen atoms in a two-dimensional (2D) plane, while the water hexamer and heptamer present a three-dimensional (3D) hydrogen bond network.^3−10^

Among the small-sized clusters, the water octamer is of particular interest. It was proposed to represent the transition to cubic structures, which dominate larger water systems and display the characteristic behavior of a solid–liquid phase transition.^11−14^ The low-energy structures of the water octamer were predicted to be nominally cubic, with the eight tricoordinated water molecules located at the corners of the cube. Such tricoordinated water molecules have also been identified on the surface of ice.^15−18^ The water octamer has thus become a superb benchmark for accurate quantification of the hydrogen bond interactions that govern the surface and bulk properties of ice.

The experimental spectroscopic characterization of the transient low-lying water cluster structures is actually a very complex task. The first detailed spectrum investigating the OH stretch region of the water octamer was obtained by Buck et al.^19^ They combined a scattering experiment with atoms and the infrared depletion technique identifying three main bands. Computational calculations to reproduce this experimental result were performed by the group of Xantheas by means of second-order vibrational perturbation theory (VPT2) to take into account anharmonicity.^20^

Recently, Li and co-authors^21^ recorded the IR spectrum of the water octamer in the high-frequency range typical of the OH stretch by means of the threshold photoionization technique using a tunable vacuum ultraviolet free electron laser (VUV-FEL), which allows for size selection of neutral clusters.^21−25^ With this new method, the spectroscopic characterization of transient low-lying structures of water clusters up to the nonamer has been feasible. The result was a more detailed spectrum than Buck’s one, and, given its complexity, the same authors tried to assign the experimental features to the five low-energy isomers of the water octamer by means of a scaled harmonic calculation of the OH stretch vibrational frequencies at the MP2/aug-cc-pvdz (avdz) level of theory.^21^ They decided to focus on the vibrational frequencies disregarding the intensities of signals in the infrared (IR) spectrum. This is because of the limitations of the experiments due to saturation effects and issues of IR absorption combined with dissociation that make a comparison between the theory and experiment based on the relative intensities of signals very difficult.

**Figure 1:**
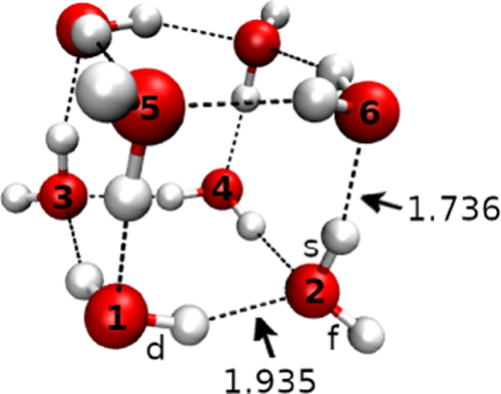
Optimized structure of conformer I (D_2d_) of (H_2_O)_8_ (O atoms are in red). Molecules 1 and 2 are representative of the ADD and AAD configurations, while Roman letters s, d, and f identify single H-donor, double H-donor, and H-donor-free OH groups, respectively. Hydrogen bond distances (in Å) are reported.

Li et al. have identified two classes of hydrogen-bonding environments in the conformer structures, according to the number of hydrogen bonds for which a monomer acts as an acceptor (A) or a donor (D). In Figure 1, water molecule 1 is in an ADD configuration since it acts as a donor toward molecules 2 and 3 while accepting a H-bond from molecule 5. In other words, it has two double H-donor OH groups. Analogously, molecule 2 acts as a donor toward molecule 6 while accepting a H-bond from molecules 1 and 4; hence, it is classified as AAD as it possesses two different OH groups named single H-donor and H-donor-free.

As noted previously,^4,16,19,26,27^ the AAD → ADD hydrogen bonds (monomer 2 → monomer 6 in Figure 1) are generally shorter than the ADD → AAD hydrogen bonds (monomer 1 → monomer 2 in Figure 1) and the corresponding frequency of the single H-donor OH stretch is typically lower than that of the double H-donor OH stretch. Consequently, Li et al. separated the IR spectrum into three regions. The highest-frequency region (around 3700 cm^–1^) is related to the H-donor-free OH groups. The intermediate region (at about 3450–3650 cm^–1^) is one of the double H-donor OH groups. Finally, the more extended low-frequency region (ranging from 3000 to 3400 cm^–1^) is characterized by the single H-donor OH stretches.

Given the complexity of the factors in play, a theoretical spectroscopic study of water clusters able to go beyond the (scaled) harmonic approximation is desirable. However, such a study is challenging for multiple reasons. First, the presence of several conformers of similar energy and separated by low barriers poses a formidable challenge to the treatment of the electronic structure. The construction of accurate force fields or *ab initio* potential energy surfaces (PESs) for water is still nowadays a hot research area due to the complexity of water systems and the need to describe accurately both the gas and condensed phase chemistry of water in a computationally affordable way.^28−38^ In this work, we employ a many-body, high-level PES, named WHBB, developed by the Bowman group.^32^ This PES takes into account 2-body interactions at the CCSD(T) level and 3-body interactions at the MP2 level of theory. Therefore, it is expected to provide a more realistic description of the water octamer than one based on an MP2 level of theory.

Secondly, the global minimum conformer is of high symmetry (D_2d_) and it is consequently characterized by a set of degenerate energy levels. For this reason, more than one conformer is expected to contribute to the experimental spectrum. Furthermore, among the five low-energy conformers, there are two enantiomers, i.e., nonsuperimposable specular systems, characterized by the C_2_ symmetry and identical spectroscopic features.

Finally, to go beyond the harmonic approximation, anharmonicity must be taken into account in a rigorous way. There are several methods, both at the classical and quantum levels, able to describe the anharmonicity of a molecular system. In particular, neutral and protonated water clusters have been investigated under numerous aspects by means of several approaches able to take anharmonicity into account. Here, we provide only a short and not exhaustive list of methods applied to the study of water clusters. For instance, classical molecular dynamics (MD) has been employed to study surface properties, local structure, and structural transitions of water clusters.^39−41^ Among methods able to reproduce nuclear quantum effects, we already mentioned the VPT2 approach, and we remind the reader that multiconfiguration time-dependent Hartree (MCTDH) provided benchmark spectroscopic calculations for the protonated water dimer,^42^ while vibrational self-consistent field (VSCF) and vibrational configuration interaction (VCI) have been successfully employed for the spectroscopy of larger water clusters.^43^ The vibrational spectroscopy of water clusters up to the 23-mer has been studied also by means of semiclassical (SC) dynamics methods, which are able to reproduce quantum effects starting from classical trajectory simulations. The main goal of these SC studies was to investigate microsolvation at the quantum dynamical level by determining the minimal water network needed for a central water molecule to be characterized by spectroscopic signals matching in frequency those of bulk water.^44,45^ Regarding the octamer, previous experimental and theoretical spectroscopic investigations have mainly involved the torsional dynamics and vibration–rotation tunneling spectra in the THz frequency range.^46,47^

In this work, we focus on the OH stretch region of the water octamer to assign the spectrum obtained by Li et al.^21^ by means of a rigorous anharmonic approach. We choose to employ the quasi-classical trajectory (QCT) technique. The reason is that QCT is a computationally affordable classical method able to provide very accurate anharmonic frequencies. Our goal is twofold: on the one hand, we want to assign the experimental spectrum, checking which conformers are actually contributing to it; on the other hand, we want to investigate the origin of the highest-frequency signal (above 3700 cm^–1^) that Li and co-workers could not explain, concluding that it was due to the presence of solvated water heptamers.

This paper is organized as follows: after describing the optimization of the cluster structures and reviewing the basic theory of QCT, we perform spectroscopy calculations on the five low-energy isomers of the water octamer employing QCT on the WHBB PES. These are presented in the Results and Discussion Section, which is ended by a detailed comparison between quasi-classical and scaled harmonic results. Finally, we recap our results and conclude the study.

## Theoretical Details

The potential energy landscape of water clusters is quite complicated and characterized by a large amount of local minima close in energy. To optimize the cluster geometry, we adopt a steepest descent algorithm with a convergence threshold on the gradient of the energy equal to 10^–6^Ha *a*_0_^–1^, i.e., we consider a geometry as optimized if |∇*V*| < 10^–6^Ha *a*_0_^–1^. With this constraint, we identify the five lowest-energy minima, with the exception of conformer V (the highest in energy among the five) for which we use a slightly higher threshold of 1.4 · 10^–6^ Ha *a*_0_^–1^. Conformers III and IV are enantiomers, and their geometries are optimized by enforcing the correct symmetry at each step of the optimization.

In Table 1, we report the relative energies of the five conformers and compare those calculated by us using the WHBB PES with those of Li et al. at the MP2 level of theory.

**Table 1:** Optimization Convergence Level (|∇*V*|) and Relative Energies of the Five Lowest-Energy Conformers of (H_2_O)_8_

conformer	|∇*V*| (Ha *a*_0_^–1^)	*E*_rel_ (WHBB; kcal mol^–1^)	*E*_rel_ (ref (21); kcal mol^–1^)
I	4.6 × 10^–7^	0.00	0.00
II	9.6 × 10^–7^	0.68	0.02
III	6.0 × 10^–7^	2.94	2.51
IV	6.0 × 10^–7^	2.94	2.51
V	1.4 × 10^–6^	4.23	2.55

**Figure 2:**
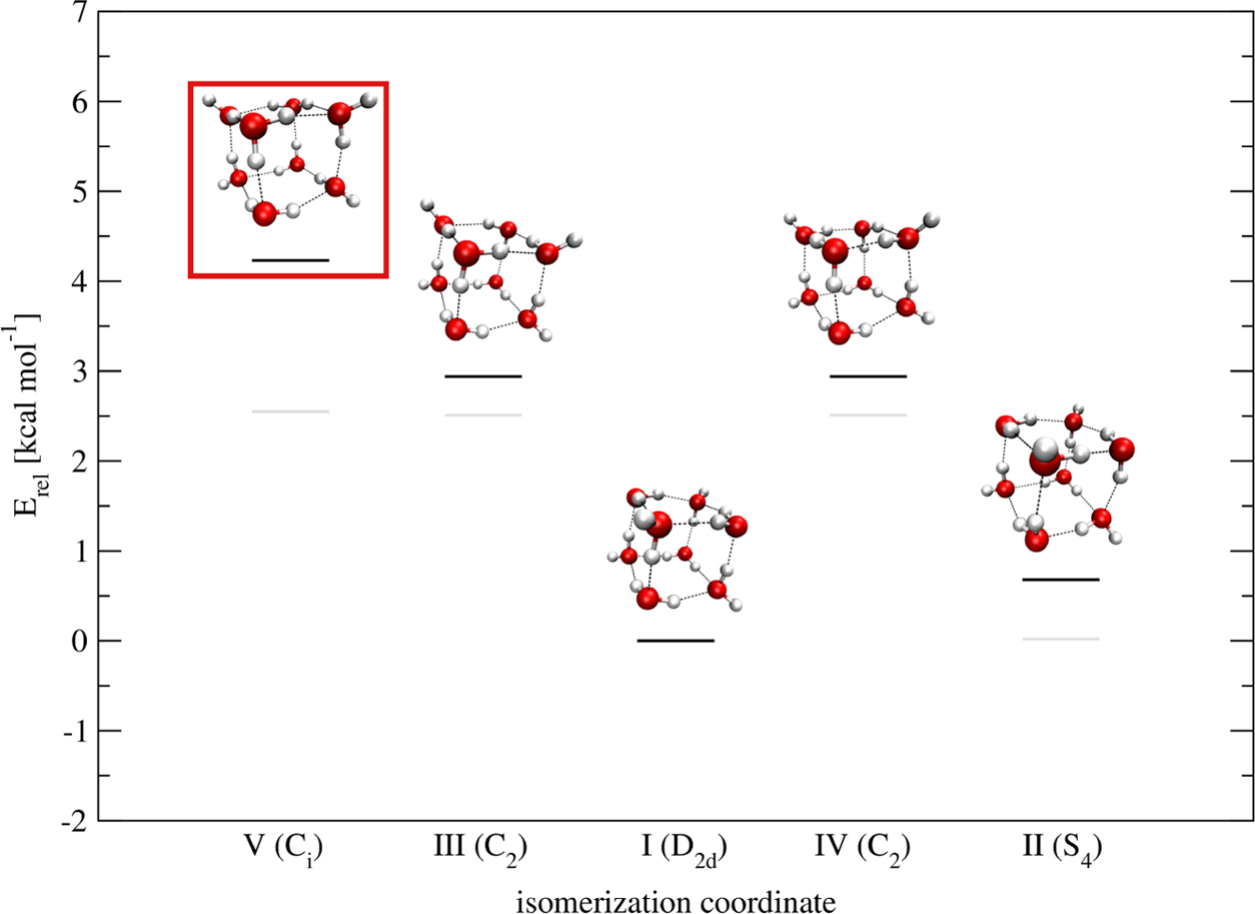
Schematic representation of the (H_2_O)_8_ PES with its five lowest-lying conformers (O atoms are in red). The energy of conformer I is set to 0. The high-energy conformer V is represented inside the rectangle. The black horizontal lines are the WHBB energies, while the gray horizontal lines refer to the MP2 energetics.

The main differences between MP2 and WHBB emerge for conformers II and V. According to WHBB, the former is no longer isoenergetic to conformer I, while the latter is energetically less accessible than what was previously predicted. Therefore, we believe conformer V is unlikely to be observed in the experiment. The geometries of the five optimized conformers and their relative energetics are reproduced in Figure 2, while coordinates associated with the conformers are available in the Supporting Information File.

Moving to the evaluation of the anharmonic frequencies of vibration, as anticipated, we employ the QCT method in our simulations. QCT simulations start the trajectories at a target and harmonically quantized energy and then evolve the trajectories in the NVE ensemble.^48^ The frequencies of vibration are obtained by means of the Fourier transform of the velocity–velocity autocorrelation function. In particular, we employ a time-averaged formulation, which has the advantage of providing better-resolved and positive-definite spectroscopic signals at the cost of a slightly longer dynamics^45^
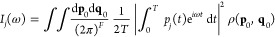
1where *j* indicates the mode under consideration, *p*_*j*_ is the associated linear momentum, and *T* is the total trajectory evolution time. ρ(**p**_0_, **q**_0_) is a distribution of initial conditions of trajectories **p**_0_ and **q**_0_ in a phase space of *F* dimensions. Therefore, eq 1 requires evolving a distribution of trajectories to evaluate the double phase space integral in a Monte Carlo fashion.

In several previous works, we have demonstrated that QCT calculations based on a single, energetically tailored trajectory are effective in the description of the classical spectroscopy of molecular species.^49−51^ An educated guess for the initial conditions consists of selecting an initial geometry (**q**_0_) corresponding to the equilibrium one (**q**_eq_) while adopting a harmonic estimate for the initial linear momenta (**p**_0_)^52^
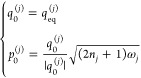
2where ω_*j*_ is the harmonic frequency of the *j*-th degree of freedom and *n*_*j*_ is its vibrational quantum number. By setting *n*_*j*_ = 0 for all degrees of freedom, the system has an initial energy equal to the harmonic zero point energy (ZPE), which is a suitable value to explore an adequate portion of the PES. The sign function in the momentum equation is essential to run symmetric trajectories for the two enantiomers, and it has been adopted also for the other isomers.

Therefore, the quasi-classical anharmonic vibrational spectrum *I*_*j*_(ω) can be computed as
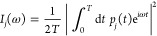
3In this work, the very accurate 4-th-order symplectic integrator developed by Brewer et al. is used.^53^ The trajectory, evolved through normal-mode dynamics, is 30,000 a.u. long and made of steps of 10 a.u. each, keeping the 6 ro-translational modes at equilibrium. To minimize energy leakage and avoid overcrowded spectra, also librational modes, which are characterized by lower frequencies compared to bendings and stretches, have been kept at equilibrium and given null initial momentum. In this way, the total energy of the simulation is below the harmonic ZPE but still high enough to guarantee an appropriate anharmonic description of the spectral features related to the OH stretches. In principle, eq 3 allows for a complete separation between the signals of different modes, which facilitates identification and assignment. In practice, when dealing with floppy and complex systems like in the case of water clusters, several signals related to coupled modes are detected in the same spectrum and a comparison between multiple spectra must be undertaken before assignment.

## Results and Discussion

### Harmonic Vibrational Analysis

Following the footsteps of Li and coauthors, we start our investigation from the harmonic frequencies. These are computed by diagonalizing the Hessian matrix at the equilibrium geometry. A central finite difference formula with a displacement of *h* = 10^–3^ a.u. is used to compute the Hessians of conformers I, II, and V. A larger displacement of *h* = 10^–2^ a.u. is adopted instead for conformers III and IV. This choice is necessary because of the numerical accuracy of the potential combined with the factor *h*^–4^ present in second derivative calculations. The choice of a displacement, which is too small, leads to tiny but undesired differences between the enantiomers and eventually to unreliable discrepancies in the QCT simulations of the two conformers. Tables 2 and 3 report the values of our harmonic frequency estimates and those at the MP2/aug-cc-pvdz level for conformer I and the two enantiomers, respectively. In this work, the focus is on the 16 stretch modes (2 per monomer), but we provide estimates also for the 8 bending modes in the Supporting Information File, where values for conformers II and V can also be found.

**Table 2:** Comparison between MP2 Harmonic (harm.) Frequencies, MP2 Scaled Harmonic (sc. harm.) Frequencies, and the Harmonic Frequencies from the WHBB PES of the 16 OH Stretch Modes of Conformer I (D_2d_) of (H_2_O)_8_^a^

mode (OH stretch)	symm	harm. (MP2)	sc. harm. (MP2)	harm. (WHBB)
ν_1_	A_1_	3225	3083	3357
ν_2_	**B_2_**	3310	3164	3373
ν_3_	**E**	3250	3107	3377
ν_4_	**E**	3250	3107	3377
ν_5_	**E**	3601	3443	3611
ν_6_	**E**	3601	3443	3611
ν_7_	**B_2_**	3620	3461	3631
ν_8_	A_1_	3618	3459	3668
ν_9_	B_1_	3667	3506	3749
ν_10_	A_2_	3668	3507	3751
ν_11_	**E**	3714	3551	3777
ν_12_	**E**	3714	3551	3777
ν_13_	**B_2_**	3879	3708	3918
ν_14_	**E**	3879	3708	3924
ν_15_	**E**	3879	3708	3924
ν_16_	A_1_	3879	3708	3933

a The symmetry species of IR-active modes are reported in bold. Data are in cm^–1^. MP2 values are taken from ref (21).

**Table 3:** Comparison between MP2 Harmonic (harm.) Frequencies, MP2 Scaled Harmonic (sc. harm.) Frequencies, and the Harmonic Frequencies from the WHBB PES of the 16 OH Stretch Modes of Conformers III and IV (C_2_) of (H_2_O)_8_^a^

mode (OH stretch)	symm	harm. (MP2)	sc. harm. (MP2)	harm. (WHBB)
ν_1_	**A**	3100	2964	3294
ν_2_	**B**	3144	3006	3304
ν_3_	**A**	3464	3312	3528
ν_4_	**B**	3469	3316	3542
ν_5_	**B**	3481	3328	3545
ν_6_	**A**	3502	3348	3558
ν_7_	**B**	3647	3487	3633
ν_8_	**A**	3661	3500	3671
ν_9_	**B**	3699	3536	3746
ν_10_	**A**	3704	3541	3754
ν_11_	**A**	3728	3564	3777
ν_12_	**B**	3765	3599	3802
ν_13_	**A**	3872	3702	3910
ν_14_	**B**	3872	3702	3911
ν_15_	**B**	3882	3711	3930
ν_16_	**A**	3882	3711	3941

a The symmetry species of IR-active modes are reported in bold. Data are in cm^–1^. MP2 values are taken from ref (21).

As expected, the two approaches yield different harmonic frequencies for the isomers. We point out that there is also an unexpected difference regarding the degeneracy of vibrational modes. We notice that the MP2 calculations for the global minimum present a 4-fold degeneracy in the 4 highest-frequency modes. However, given the symmetry point group of conformer I (D_2d_), at maximum doubly degenerate energy levels are allowed, which is what we indeed get from our optimization. Something similar happens for the two enantiomers (and conformers II and V in the Supporting Information File). In the case of conformers III and IV, the symmetry point group is C_2_, which permits to have only nondegenerate energy levels. Conversely, the MP2 calculations show doubly degenerate modes, while in our case, the correct nondegeneracy of all modes is respected. Li and coauthors point out in their paper that unwanted degeneracies are removed when looking at the IR intensities.

**Figure 3:**
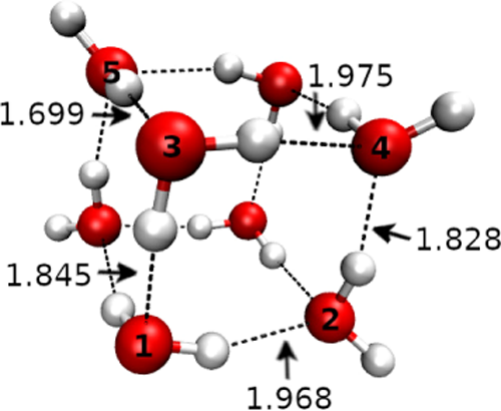
Optimized structure of conformer III (C_2_) of (H_2_O)_8_ (O atoms are in red). Hydrogen bond distances (in Å) are reported.

The symmetry issue involves the H-donor-free OH stretches of all isomers (modes ν_13_, ν_14_, ν_15_, ν_16_) and the double H-donor antisymmetric OH stretches of isomer V (modes ν_9_, ν_10_). This leads to the recognition of a reduced number of energy levels, which may jeopardize the spectroscopic assignment. Furthermore, from what Li et al. reported in their article, our understanding is that they suggest that, while conformers I and II possess four single H-donor, eight double H-donor, and four H-donor-free OH stretches, conformers III–V show six single H-donor and six double H-donor OH stretches. However, we notice that since each conformer has four AAD and four ADD monomers, the number of single and double H-donor OH stretches is four and eight for all conformers. The difference between the two sets of conformers lies instead in the fact that conformers III–V exhibit two new types of hydrogen bonds, AAD → AAD H-bonds (monomer 2 → monomer 4 in Figure 3) and ADD → ADD H-bonds (monomer 3 → monomer 1 in Figure 3), which are comparable in length. Hence, the harmonic vibrational frequencies of single H-donor OH stretches involved in AAD → AAD H-bonds (modes ν_3_, ν_4_) are close to those of double H-donor OH stretches involved in ADD → ADD H-bonds (modes ν_5_, ν_6_). Eventually, for both MP2 and WHBB, the harmonic values lie far from the experimental signals, so anharmonicities must be taken into account. For this reason, we move to present our QCT results.

### QCT Assignment of the Water Octamer Spectrum

**Figure 4:**
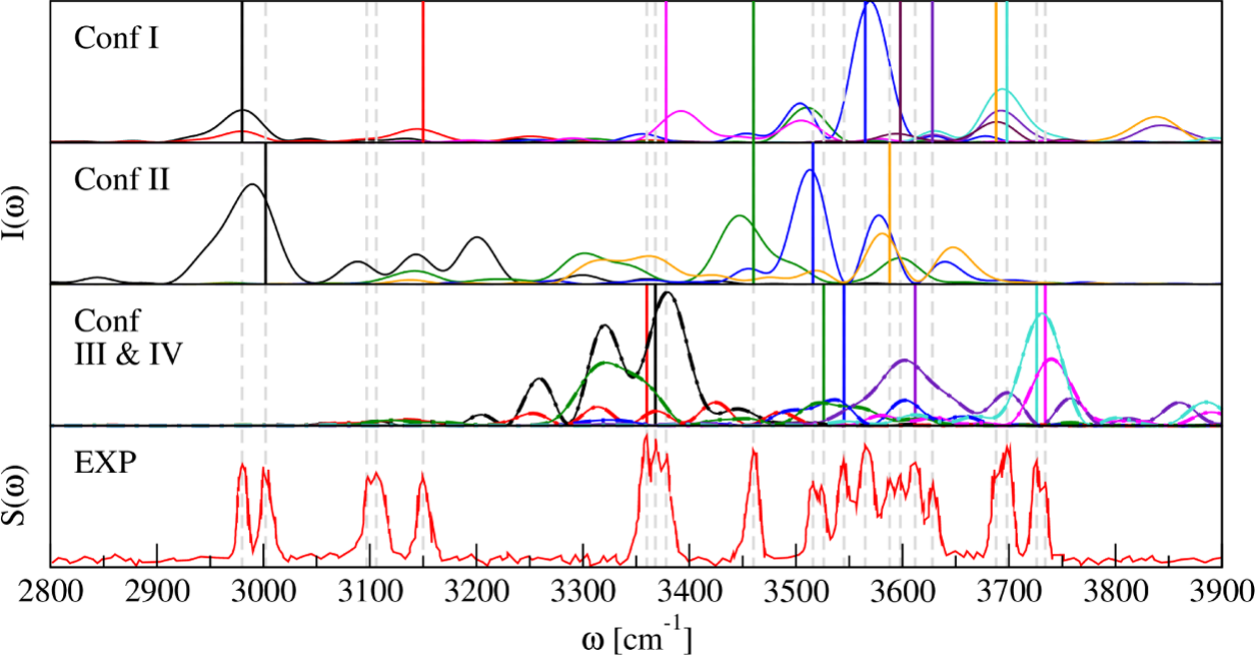
Comparison between OH stretch vibrational frequencies of the experimental IR spectrum (EXP) and simulated QCT power spectra of conformers I–IV. Vertical lines represent the experimental frequencies. The different colors within a panel indicate the simulation of different modes. The same colors in different panels are not related. Straight and dashed lines in the third panel correspond to conformers III and IV, respectively. *S*(ω) indicates the IR absorbance. The experimental spectrum is reproduced from ref (21) (https://creativecommons.org/licenses/by/4.0/).

Figure 4 shows the power spectra obtained for the 4 lowest-lying conformers by means of the QCT simulations. Conformer V lies higher in energy, and from our QCT study, it turns out that it is not contributing to the spectrum. Only the modes contributing to the experimental spectrum are displayed. QCT frequencies of all modes (conformer V included) are reported in the Supporting Information File.

Figure 4 demonstrates that we are able to reproduce the main features of the IR spectrum by means of our QCT simulations. In general, the QCT spectra present several features in addition to the signal related to the target mode. This is mainly due to the coupling between different modes or seldom it could be due to finite time propagation leading to spurious classical resonances. To perform a close comparison to the experiment, we extrapolate the peaks at 3097, 3360, 3368, 3688, 3734 cm^–1^, and in the region 3526–3628 cm^–1^ through a digitalization tool.

In the case of conformer I, we locate signals at 2980, 3144, 3392, 3445, 3570, 3595, 3629, 3687, and 3694 cm^–1^, which we relate to the experimental signals observed at 2980, 3150, 3378, 3460, 3565, 3598, 3628, 3688, and 3698 cm^–1^. In the simulated spectrum of conformer II, the calculated features at 2990, 3447, 3513, and 3581 cm^–1^ are in good agreement with the observed signals at 3002, 3460, 3516, and 3588 cm^–1^. Moving to conformers III and IV, i.e., the two enantiomers, the spectroscopic features found by means of QCT at 3369, 3379, 3523, 3536, 3602, 3722, and 3731 cm^–1^ could be responsible for the observed signals at 3360, 3368, 3526, 3545, 3612, 3726, and 3734 cm^–1^. The spectra of the two conformers were obtained running independent trajectories, with symmetric initial conditions, and they are perfectly superimposable, as depicted in Figure 4.

We notice that by means of our QCT procedure, we have not assigned the two experimental signals located at 3097 and 3106 cm^–1^. One possibility is that they are related to two bending combinations at 3103 and 3110 cm^–1^, respectively. To the best of our knowledge, there is no experimental spectrum recorded for the bendings of the water octamer, so we cannot make an assignment of the bending region. However, we performed QCT calculations also for the bendings and reported the detailed results in the Supporting Information File.

### Comparison between Scaled Harmonic and QCT Assignments

A comparison between QCT and scaled harmonic frequencies points out the limitations of the *ad hoc* scaling procedure. Table 4 summarizes the results.

**Table 4:** Comparison between Experimental (exp), MP2/avdz Scaled Harmonic (sc. harm.), and QCT Frequencies Based on the WHBB PES^a^

exp^b^	sc. harm.-MP2^c^	mode-MP2	QCT-WHBB	mode-WHBB
**2980**	**2964(III and IV)**	ν_1_	2980(I)	ν_4_
	**2971(V)**	ν_1_		
**3002**	**3006(III and IV)**	ν_2_	2990(II)	ν_3_
	**2994(V)**	ν_2_		
3097	3104(II)	ν_4_		
**3106**	**3107(I)**	ν_3_, ν_4_		
**3150**	**3164(I)**	ν_2_	3144(I)	ν_2_
3360	3312(III and IV)	ν_3_	3369(III and IV)	ν_6_
	3316(III and IV)	ν_4_		
	3311(V)	ν_3_		
3368	3328(III and IV)	ν_5_	3379(III and IV)	ν_4_
**3378**	**3348(III and IV)**	ν_6_	3392 (I)	ν_7_
	**3346(V)**	ν_5_		
**3460**	**3443(I)**	ν_5_, ν_6_	3445(I)	ν_6_
	**3461(I)**	ν_7_	3447(II)	ν_5_
	**3444(II)**	ν_5_		
	**3450(II)**	ν_6_, ν_7_		
**3516**	3500(III and IV)	ν_8_	3513(II)	ν_11_
3526	3531(II)	ν_10_, ν_11_	3523(III and IV)	ν_9_
3545	3548(II)	ν_12_	3536(III and IV)	ν_8_
	3536(III and IV)	ν_9_		
	3541(III and IV)	ν_10_		
3565	3551(I)	ν_11_, ν_12_	3570(I)	ν_11_
3588	3587(V)	ν_12_	3581(II)	ν_12_
3598	3599(III and IV)	ν_12_	3595(I)	ν_12_
3612			3602(III and IV)	ν_13_
**3628**			3629(I)	ν_13_
3688	3702(III and IV)	ν_13_, ν_14_	3687(I)	ν_15_
	3702(V)	ν_13_, ν_14_		
**3698**	**3708(I)**	ν_13_, ν_14_, ν_15_	3694(I)	ν_14_
	**3708(II)**	ν_13_, ν_14_, ν_15_		
**3726**	**heptamer**		3722(III and IV)	ν_14_
3734	heptamer		3731(III and IV)	ν_16_
MAE	12.6		6.2	

a The conformer responsible for the signal is indicated in parentheses. Data are in cm^–1^. Scaled harmonic values are taken from ref (21).

b Bold values are from ref (21); the others have been extrapolated through a digitalization tool.

c Assignments in bold are those directly assigned in ref (21); the other frequencies have been taken from ref (21) and assigned in a way to minimize the MAE.

**Figure 5:**
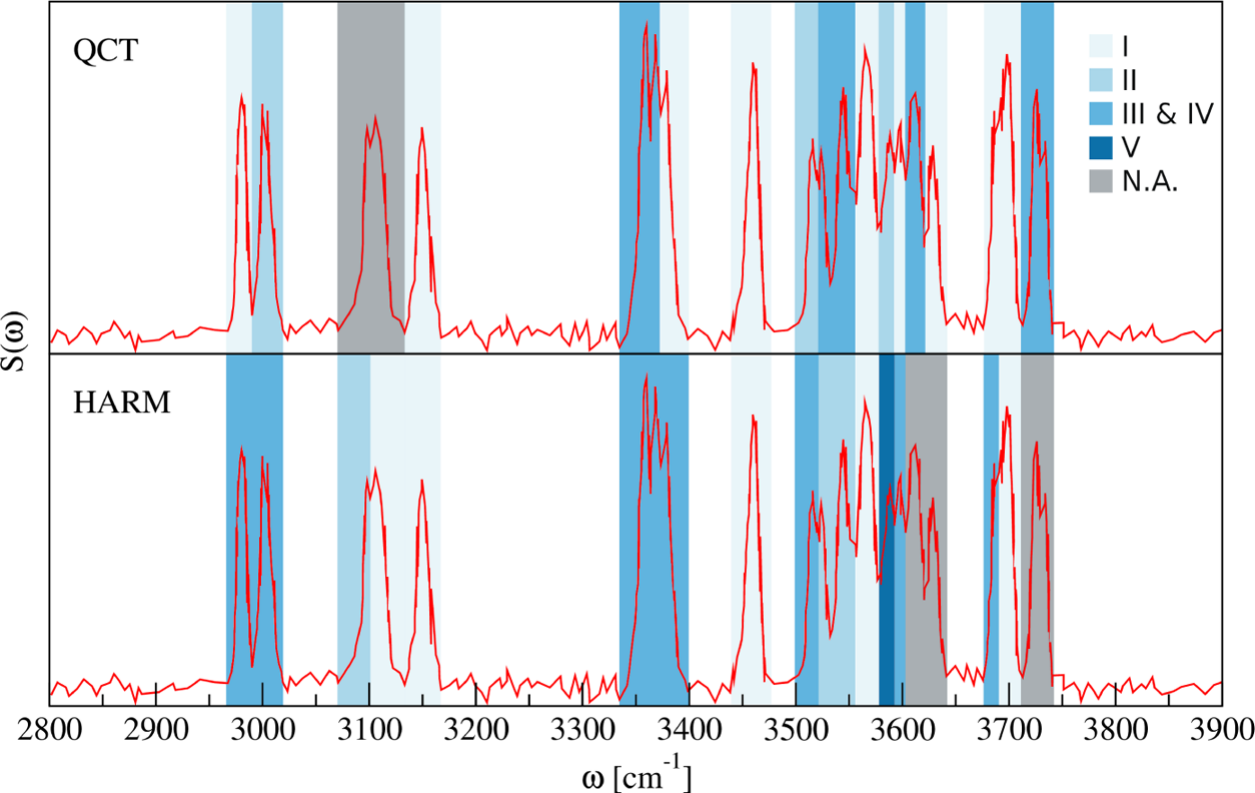
Comparison between QCT and scaled harmonic assignment of the OH stretch region of the water octamer. The red line is the experimental spectrum, reproduced from ref (21) (https://creativecommons.org/licenses/by/4.0/). Signals unassigned or assigned to the solvated heptamer are in gray.

First of all, the scaling parameter shifts the harmonic frequencies of all modes by the same relative amount (≈5%), while running a QCT dynamics on the PES allows us to account for the deviations from the harmonic approximation mode by mode, showing variations in the range between 2 and 10%. Second, the mean absolute error (MAE) is substantially reduced when employing a QCT simulation. To compute the MAE of the scaled harmonic approach, we have employed not only the set of frequencies assigned by the authors in ref (21) but also all other frequencies reported therein, assigning them according to their proximity to the experimental values. In this way, the scaled harmonic approach is characterized by an MAE of about 13 cm^–1^, which is lowered to about 6 cm^–1^ in the case of QCT. Third, QCT offers a new insight into the three regions of the spectrum assigned to single H-donor, double H-donor, and H-donor-free OH groups. The H-donor-free region is predicted to extend down to 3600 cm^–1^, while the triple peak at around 3370 cm^–1^ contains both single and double H-donor modes. Moreover, the scaled harmonic approach is not able to assign 4 spectroscopic features, and in two cases, the presence of the solvated heptamer is invoked, while by means of QCT, we can assign the whole spectral range of the OH stretches with the exception of two signals, which could be related to bending combinations. Finally, QCT is capable of assigning the signals to the lower-energy isomers, as depicted by the color map in Figure 5. We do not need conformer V to make our spectral assignment, which is consistent with the high relative energy of this conformer.

## Summary and Conclusions

We have presented a quasi-classical trajectory study of the ice–precursor water octamer, for which a recent experiment has been reported.^21^ Our primary goal was to come up with a more rigorous assignment than the original one based on a scaled harmonic approach at the MP2 level of theory. This was necessary because there were some unassigned spectroscopic features in the original work, while others were explained by invoking the presence of the solvated water heptamer. Another assignment of the water octamer OH stretch spectrum was performed a few years ago by the group of Xantheas,^20^ though the available reference experiment was not recent and much less detailed than the one we refer to.

We employed the WHBB PES in our calculations. This is a robust many-body PES, which has been widely used in spectroscopic studies of water systems. The construction of high-level water PESs is a very active field in theoretical chemistry given the importance of water in several research fields. Examples of other PESs adopting a many-body theory include MB-pol from the Paesani group^34^ and q-AQUA from the Bowman group.^54^ These PESs are still being evolved to come up with a high-level description of both gas and condensed phases of water. Indeed, at the time of writing this paper, two new PESs, named MB-pol(2023) and q-AQUA-pol, appeared in the literature.^55,56^

Xantheas recently performed benchmark calculations for a large set of water potentials based on the energetics of several water clusters.^57^ Conformers I and II of the water octamer were included in these calculations, and it turned out that WHBB returns a larger energy gap exceeding by about 0.4 kcal mol^–1^ the one calculated with MB-pol and q-AQUA. This is not an issue for our calculations for two reasons: first of all, it is shown that, in comparison to benchmarks, WHBB is overall a very accurate PES. Then, in our analysis, we assigned signals to conformer II and went up to the two enantiomers, which are higher in energy, so we deem we have not overlooked any spectroscopic feature related to conformer II.

The use of QCT and the WHBB PES led us to assign the OH stretch region of the spectrum of the water octamer without the need to invoke the presence of either the water heptamer or conformer V. This assignment is a difficult task since the OH stretch region extends more than 700 cm^–1^ and some spectroscopic features are very anharmonic. We did not assign two signals at around 3100 cm^–1^ because we think they are due to bending combinations. To better characterize them, we should employ a semiclassical approach^58−69^ able to reproduce anharmonic overtones and combination bands, which is beyond the scope of this paper.

In our assignment, we took into account the symmetry of conformers to identify expected degeneracies and determine which modes are IR-active and therefore which modes can contribute to the spectrum. However, trajectories have evolved in full dimensionality and IR intensities have not been considered given the experimental issues anticipated in the Introduction section, which make them not fully reliable.

At the request of an anonymous reviewer, we further investigated the reliability of our WHBB-based calculations in comparison to MB-pol- and q-AQUA-based ones. Since WHBB is expected to perform worse than the newest potentials for the high-energy isomers, we compared the energetics of conformer V for the three different potentials. We found that, as anticipated, WHBB overestimates the energy of conformer V. However, conformer V is still the highest-energy conformer also upon calculations employing MB-pol or q-AQUA. Therefore, from the energetic point of view, our conclusions are unchanged. Furthermore, MP2/avdz calculations performed by Li^21^ show that the interconversion between conformers III and V is kinetically disadvantaged due to a high barrier (about 8 kcal mol^–1^). These facts strengthen our conclusion that conformer V is not contributing to the experimental spectrum.

Then, we calculated the QCT spectra for the 5 conformers to check for spectroscopic differences. We notice that for conformers I–IV the spectra are similar for the three potentials, while more differences are found in the spectrum of conformer V. This was expected due to the energy difference among PESs, but no remarkable difference is found in the 3100 cm^–1^ region, which is where QCT is not assigning signals related to fundamentals. An interesting consideration is that, as anticipated in the Theoretical Details Section, floppy systems like the octamer present several coupled modes; hence, frequencies are sometimes swapped between modes for the three PESs. The largest differences in the QCT estimates are detected for the modes where there is a large difference already in the harmonic frequency. For these reasons, our final conclusion is that, relatively to the calculations reported in this paper, the use of WHBB provides results of equal quality to employing the more recent MB-pol and q-AQUA PESs. Energetics, harmonic, and QCT frequencies (of the experimentally detected modes) for MB-pol and q-AQUA PESs are reported in the Supporting Information File.

The water octamer is a supramolecular system characterized by 66 vibrational degrees of freedom. Of these, 8 are bendings, 16 are stretchings, and the remaining 42 are frustrated rotations and translations. Furthermore, there is a narrow energy gap between several conformers and we found 4 of them contributing to the spectrum. We are not aware of experiments concerning the bendings of the water octamer. For this reason, we could not perform an assignment on the bending region of the spectrum. Our theoretical results for the bendings of the main conformers are reported in the Supporting Information File.

## Supporting Information Available

Coordinates and energy of equilibrium configurations for conformers I–V of (H_2_O)_8_; harmonic and QCT frequencies from the WHBB PES of OH stretch and bending modes of conformers I–V; MP2 harmonic frequencies of OH stretch modes of conformers II and V from ref (21); and energetics, harmonic, and QCT frequencies (of the experimentally detected modes) for MB-pol and q-AQUA PESs (PDF)
