# Supplementary material for: Anharmonic Assignment of the Water Octamer Spectrum in the OH Stretch Region
Source: J Phys Chem A. 2023 Jul 21;127(30):6213–21. doi: 10.1021/acs.jpca.3c02902 (PMC10405218; doi:10.1021/acs.jpca.3c02902)
Supplement: Supplementary file 1 — jp3c02902_si_001.pdf [file jp3c02902_si_001.pdf]

Supplementary information for Anharmonic assignment of the water octamer spectrum in the OH stretch region

Davide Barbiero,[†] Gianluca Bertaina,[‡] Michele Ceotto,[†] and Riccardo Conte[*,†]

[†]*Dipartimento di Chimica, Università degli Studi di Milano, via Golgi 19, 20133 Milano (Italy)*

[‡]*Istituto Nazionale di Ricerca Metrologica, Strada delle Cacce 91, I-10135 Torino (Italy)*

E-mail: riccardo.conte1@unimi.it

Coordinates and energy of low-energy minima of $(H_2O)_8$ on the WHBB PES

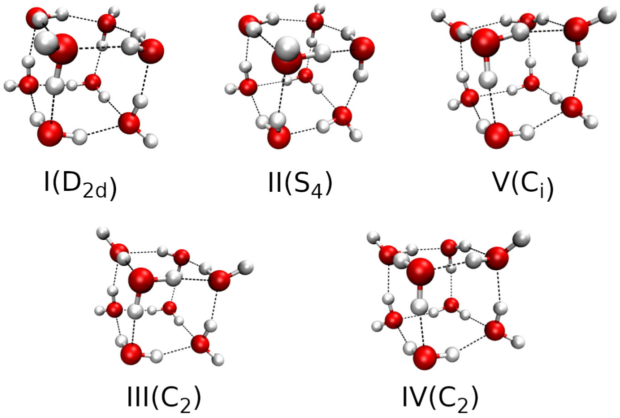

Figure S1: Optimized structures of the five conformers of $(H_2O)_8$ (O atoms are in red). Point group symmetries are noted in parenthesis.

Table S1: Structure and energy of Conformer I of $(H_2O)_8$. Energy is in Hartree, while atom positions are in Å.

atom	E=-0.11626467		
H	-1.4287299694	0.7643767115	1.5343955705
H	-1.4287436346	-0.7643704961	1.5343725481
H	0.0000329733	2.1750681891	0.3841037323
H	0.0000565570	2.9381418758	1.7307461300
H	0.0000070704	-2.9381695216	1.7306513861
H	-0.0000013629	-2.1750516888	0.3840343431
H	-2.1750552726	0.0000361506	-0.3840653057
H	-2.9381668543	0.0000573687	-1.7306860488
H	0.7643574613	-1.4287311460	-1.5344186102
H	-0.7643900330	-1.4287210473	-1.5344018418
H	0.7643768542	1.4287976703	-1.5343753493
H	-0.7643706729	1.4288073472	-1.5343587679
H	2.1750704465	0.0000036930	-0.3841135545
H	2.9381497278	0.0000195395	-1.7307525105
H	1.4287885857	-0.7643961982	1.5343404325
H	1.4288023427	0.7643510010	1.5343648836
O	-1.9890768423	0.0000109931	1.3418348399
O	0.0000455402	2.0571933765	1.3600665961
O	0.0000089796	-2.0572087125	1.3600011338
O	-2.0572078394	0.0000483828	-1.3600315210
O	-0.0000178661	-1.9890670224	-1.3418641086
O	0.0000087503	1.9891370419	-1.3418024571
O	2.0571995640	0.0000206556	-1.3600769059
O	1.9891315344	-0.0000245418	1.3417918098

Table S2: Structure and energy of Conformer II of $(H_2O)_8$. Energy is in Hartree, while atom positions are in Å.

atom	E=-0.11517894		
H	-0.7525760578	1.3966942522	1.5462420398
H	0.0026204840	2.1839856844	0.4753832963
H	-1.3552219113	-0.8191530302	1.5380960544
H	-2.6886866686	-0.2458288647	2.0802926897
H	1.3551927267	0.8191931745	1.5380793188
H	2.6886561547	0.2458792207	2.0802901706
H	0.7525467713	-1.3966527075	1.5462719818
H	-0.0026489649	-2.1839693946	0.4754315480
H	-2.1839894314	0.0026332787	-0.4753850972
H	-1.3966847439	-0.7525746828	-1.5462259405
H	2.1839586004	-0.0026315783	-0.4753816152
H	1.3966541342	0.7525532661	-1.5462395089
H	-0.8191889042	1.3551968145	-1.5380789158
H	-0.2458745277	2.6886578511	-2.0802952007
H	0.8191589304	-1.3552175993	-1.5380481195
H	0.2458435603	-2.6886878774	-2.0802398915
O	0.0412553711	1.9349734389	1.4090892918
O	-1.9904454637	-0.0718410499	1.4510241054
O	1.9904159970	0.0718790058	1.4510238949
O	-0.0412838332	-1.9349363757	1.4091319107
O	-1.9349663878	0.0412577805	-1.4090885960
O	1.9349363667	-0.0412757087	-1.4090845467
O	-0.0718779390	1.9904235795	-1.4510213793
O	0.0718472846	-1.9904419884	-1.4509789295

Table S3: Structure and energy of Conformer III of $(H_2O)_8$. Energy is in Hartree, while atom positions are in Å.

atom	E=-0.11158907		
H	-0.5481739640	-1.5880424085	-1.5323674450
H	0.9572684881	-1.6058930256	-1.3054773976
H	2.9347806175	-1.7721365173	0.4455293829
H	2.2317005520	-0.4123109498	0.2870201211
H	0.5525506626	-1.5792677249	1.5409836966
H	-0.9528495551	-1.6014307444	1.3141943020
H	-2.9299949339	-1.7805404345	-0.4359795957
H	-2.2297961577	-0.4184603600	-0.2842114429
H	0.3090185763	0.4250514674	-2.1024161027
H	-0.5423749964	1.5451728770	-1.5000699631
H	-2.6993156196	2.0376528693	-0.0888150064
H	-1.4490559547	1.4798031745	0.6381981098
H	-0.3088889807	0.4348051048	2.1010226280
H	0.5401358068	1.5537239695	1.4931173093
H	2.6960304679	2.0438006560	0.0794370396
H	1.4469635214	1.4797063331	-0.6448029870
O	0.2915784322	-1.4157138716	-1.9784509436
O	2.0754128767	-1.3735383079	0.3123150379
O	-0.2875665608	-1.4065077178	1.9862123121
O	-2.0714757902	-1.3794690047	-0.3047318150
O	0.3167941110	1.3794278246	-1.9090896954
O	-2.0005432880	1.3920734704	-0.1761549232
O	-0.3186827567	1.3881911130	1.9029585398
O	1.9986289872	1.3971800550	0.1699731362

Table S4: Structure and energy of Conformer IV of $(H_2O)_8$. Energy is in Hartree, while atom positions are in Å.

atom	E=-0.11158907		
H	0.5525506626	-1.5792677249	-1.5409836966
H	-0.9528495551	-1.6014307444	-1.3141943020
H	-2.9299949339	-1.7805404345	0.4359795957
H	-2.2297961577	-0.4184603600	0.2842114429
H	0.5401358068	1.5537239695	-1.4931173093
H	-0.3088889807	0.4348051048	-2.1010226280
H	2.6960304679	2.0438006560	-0.0794370396
H	1.4469635214	1.4797063331	0.6448029870
H	-0.5481739640	-1.5880424085	1.5323674450
H	0.9572684881	-1.6058930256	1.3054773976
H	-1.4490559547	1.4798031745	-0.6381981098
H	-2.6993156196	2.0376528693	0.0888150064
H	2.9347806175	-1.7721365173	-0.4455293829
H	2.2317005520	-0.4123109498	-0.2870201211
H	0.3090185763	0.4250514674	2.1024161027
H	-0.5423749964	1.5451728770	1.5000699632
O	-0.2875665608	-1.4065077178	-1.9862123121
O	-2.0714757902	-1.3794690047	0.3047318150
O	-0.3186827567	1.3881911130	-1.9029585398
O	1.9986289872	1.3971800550	-0.1699731362
O	0.2915784322	-1.4157138716	1.9784509436
O	-2.0005432880	1.3920734704	0.1761549232
O	2.0754128767	-1.3735383079	-0.3123150379
O	0.3167941110	1.3794278246	1.9090896954

Table S5: Structure and energy of Conformer V of $(H_2O)_8$. Energy is in Hartree, while atom positions are in Å.

atom	E=-0.10955586		
H	2.1973160640	-0.57401974517	0.2381605627
H	2.8068306092	-1.9871159495	0.3190909731
H	0.4317263864	-1.6441896372	1.4649513938
H	-0.3261961986	-0.4710330009	2.0898679970
H	-2.8369052028	-1.8433113119	-0.1301528631
H	-1.5474800291	-1.3920153904	0.6001212761
H	0.8343072604	-1.6112215563	-1.3776741092
H	-0.6712870515	-1.4939079843	-1.5727031393
H	-2.1974148737	0.5740197010	-0.2380150445
H	-2.8069264787	1.9871164991	-0.3189579634
H	-0.4318203532	1.6441875903	-1.4648090551
H	0.3261015592	0.4710308846	-2.0897241218
H	1.5473780473	1.3920141274	-0.5999688622
H	2.8367958300	1.8433160168	0.1303148099
H	-0.8344154330	1.6112215650	1.3778239888
H	0.6711785425	1.4939089009	1.5728540239
O	1.9749122844	-1.5238123067	0.2233019299
O	-0.4171832474	-1.4145400112	1.8626328030
O	-2.0949821014	-1.2468665447	-0.2087590564
O	0.1677710320	-1.3696566190	-2.0360093141
O	-1.9750094815	1.5238120018	-0.2231614854
O	0.4170902215	1.4145375222	-1.8624882460
O	2.0948755368	1.2468669270	0.2089149071
O	-0.1678797798	1.3696555190	2.0361592988

Vibrational frequencies of the OH stretch modes of Conformers I - V

Table S6: Comparison between MP2 harmonic (Harm.) frequencies, MP2 scaled harmonic (Sc. harm.) frequencies, harmonic and QCT anharmonic frequencies from the WHBB PES of the 16 OH stretch modes of Conformer I (D_{2d}) of $(H_2O)_8$. The symmetry species of IR-active modes are reported in bold. Data are in cm^{-1}. MP2 values are taken from Ref. 1.

Mode (OH stretch)	Symm	Harm. (MP2)	Sc. harm. (MP2)	Harm. (WHBB)	QCT (WHBB)
ν_1	A_1	3225	3083	3357	3234
ν_2	$\mathbf{B_2}$	3310	3164	3373	3144
ν_3	**E**	3250	3107	3377	2984
ν_4	**E**	3250	3107	3377	2980
ν_5	**E**	3601	3443	3611	3440
ν_6	**E**	3601	3443	3611	3445
ν_7	$\mathbf{B_2}$	3620	3461	3631	3392
ν_8	A_1	3618	3459	3668	3464
ν_9	B_1	3667	3506	3749	3582
ν_{10}	A_2	3668	3507	3751	3517
ν_{11}	**E**	3714	3551	3777	3570
ν_{12}	**E**	3714	3551	3777	3595
ν_{13}	$\mathbf{B_2}$	3879	3708	3918	3629
ν_{14}	**E**	3879	3708	3924	3694
ν_{15}	**E**	3879	3708	3924	3687
ν_{16}	A_1	3879	3708	3933	3826

Table S7: Comparison between MP2 harmonic (Harm.) frequencies, MP2 scaled harmonic (Sc. harm.) frequencies, harmonic and QCT anharmonic frequencies from the WHBB PES of the 16 OH stretch modes of Conformer II (S_4) of $(H_2O)_8$. The symmetry species of IR-active modes are reported in bold. Data are in cm^{-1}. MP2 values are taken from Ref. 1.

Mode (OH stretch)	Symm	Harm. (MP2)	Sc. harm. (MP2)	Harm. (WHBB)	QCT (WHBB)
ν_1	A	3222	3080	3323	3119
ν_2	**E**	3275	3131	3346	2995
ν_3	**E**	3275	3131	3346	2990
ν_4	**B**	3247	3104	3355	3078
ν_5	**B**	3603	3444	3612	3447
ν_6	**E**	3609	3450	3615	3414
ν_7	**E**	3609	3450	3615	3428
ν_8	A	3619	3460	3661	3509
ν_9	A	3666	3505	3735	3602
ν_{10}	**E**	3694	3531	3749	3532
ν_{11}	**E**	3694	3531	3749	3513
ν_{12}	**B**	3711	3548	3759	3581
ν_{13}	**E**	3879	3708	3921	3869
ν_{14}	**E**	3879	3708	3921	3862
ν_{15}	**B**	3879	3708	3924	3642
ν_{16}	A	3879	3708	3932	3712

Table S8: Comparison between MP2 harmonic (Harm.) frequencies, MP2 scaled harmonic (Sc. harm.) frequencies, harmonic and QCT anharmonic frequencies from the WHBB PES of the 16 OH stretch modes of Conformers III and IV (C_2) of $(H_2O)_8$. The symmetry species of IR-active modes are reported in bold. Data are in cm^{-1}. MP2 values are taken from Ref. 1.

Mode (OH stretch)	Symm	Harm. (MP2)	Sc. harm. (MP2)	Harm. (WHBB)	QCT (WHBB)
ν_1	**A**	3100	2964	3294	2958
ν_2	**B**	3144	3006	3304	2843
ν_3	**A**	3464	3312	3528	3282
ν_4	**B**	3469	3316	3542	3379
ν_5	**B**	3481	3328	3545	3310
ν_6	**A**	3502	3348	3558	3369
ν_7	**B**	3647	3487	3633	3481
ν_8	**A**	3661	3500	3671	3536
ν_9	**B**	3699	3536	3746	3523
ν_{10}	**A**	3704	3541	3754	3558
ν_{11}	**A**	3728	3564	3777	3645
ν_{12}	**B**	3765	3599	3802	3692
ν_{13}	**A**	3872	3702	3910	3602
ν_{14}	**B**	3872	3702	3911	3722
ν_{15}	**B**	3882	3711	3930	3740
ν_{16}	**A**	3882	3711	3941	3731

Table S9: Comparison between MP2 harmonic (Harm.) frequencies, MP2 scaled harmonic (Sc. harm.) frequencies, harmonic and QCT anharmonic frequencies from the WHBB PES of the 16 OH stretch modes of Conformer V (C_i) of $(H_2O)_8$. The symmetry species of IR-active modes are reported in bold. Data are in cm^{-1}. MP2 values are taken from Ref. 1.

Mode (OH stretch)	Symm	Harm. (MP2)	Sc. harm. (MP2)	Harm. (WHBB)	QCT (WHBB)
ν_1	A_g	3108	2971	3289	2978
ν_2	$\mathbf{A_u}$	3132	2994	3298	2977
ν_3	$\mathbf{A_u}$	3463	3311	3507	3414
ν_4	A_g	3471	3318	3512	3395
ν_5	$\mathbf{A_u}$	3500	3346	3539	3208
ν_6	A_g	3479	3326	3550	3242
ν_7	$\mathbf{A_u}$	3657	3496	3640	3471
ν_8	A_g	3651	3490	3652	3556
ν_9	A_g	3698	3535	3751	3442
ν_{10}	$\mathbf{A_u}$	3698	3535	3753	3507
ν_{11}	A_g	3746	3581	3772	3601
ν_{12}	$\mathbf{A_u}$	3752	3587	3775	3651
ν_{13}	$\mathbf{A_u}$	3872	3702	3905	3700
ν_{14}	A_g	3872	3702	3908	3699
ν_{15}	$\mathbf{A_u}$	3882	3711	3931	3766
ν_{16}	A_g	3882	3711	3941	3767

Vibrational frequencies of the OH bending modes of Conformers I - V

Table S10: Comparison between harmonic (Harm.) and QCT anharmonic frequencies from the WHBB PES of the 8 OH bending modes of Conformer I (D_{2d}) of $(H_2O)_8$. The symmetry species of IR-active modes are reported in bold. Data are in cm^{-1}.

Mode (OH bending)	Symm	Harm.	QCT
ν_1	**E**	1666	1670
ν_2	**E**	1666	1666
ν_3	$\mathbf{B_2}$	1667	1558
ν_4	A_1	1686	1583
ν_5	$\mathbf{B_2}$	1693	1653
ν_6	**E**	1695	1630
ν_7	**E**	1696	1659
ν_8	A_1	1704	1740

Table S11: Comparison between harmonic (Harm.) and QCT anharmonic frequencies from the WHBB PES of the 8 OH bending modes of Conformer II (S_4) of $(H_2O)_8$. The symmetry species of IR-active modes are reported in bold. Data are in cm^{-1}.

Mode (OH bending)	Symm	Harm.	QCT
ν_1	**B**	1662	1529
ν_2	**E**	1666	1543
ν_3	**E**	1666	1548
ν_4	A	1687	1656
ν_5	A	1695	1577
ν_6	**E**	1714	1710
ν_7	**E**	1714	1725
ν_8	**B**	1748	1696

Table S12: Comparison between harmonic (Harm.) and QCT anharmonic frequencies from the WHBB PES of the 8 OH bending modes of Conformers III and IV (C_2) of $(H_2O)_8$. The symmetry species of IR-active modes are reported in bold. Data are in cm^{-1}.

Mode (OH bending)	Symm	Harm.	QCT
ν_1	**B**	1676	1532
ν_2	**B**	1677	1662
ν_3	**A**	1679	1590
ν_4	**A**	1684	1645
ν_5	**B**	1685	1646
ν_6	**A**	1697	1674
ν_7	**B**	1729	1682
ν_8	**A**	1734	1684

Table S13: Comparison between harmonic (Harm.) and QCT anharmonic frequencies from the WHBB PES of the 8 OH bending modes of Conformer V (C_i) of $(H_2O)_8$. The symmetry species of IR-active modes are reported in bold. Data are in cm^{-1}.

Mode (OH bending)	Symm	Harm.	QCT
ν_1	$\mathbf{A_u}$	1667	1572
ν_2	A_g	1680	1556
ν_3	$\mathbf{A_u}$	1683	1598
ν_4	A_g	1686	1631
ν_5	A_g	1708	1658
ν_6	$\mathbf{A_u}$	1708	1668
ν_7	A_g	1742	1727
ν_8	$\mathbf{A_u}$	1747	1675

Comparison of the energetics of Conformers I - V for the four levels of theory adopted in the work

Table S14: Relative energies, in kcal mol^{-1}, of the five lowest-energy conformers of $(H_2O)_8$ for the four levels of theory adopted in the work.

Conf.	MP2 (Ref. 1)	WHBB	q-AQUA	MB-pol
I	0.00	0.00	0.00	0.00
II	0.02	0.68	0.38	0.29
III	2.51	2.94	2.83	2.81
IV	2.51	2.94	2.83	2.81
V	2.55	4.23	3.06	3.17

Comparison of the vibrational frequencies of the experimentally detected OH stretch modes of Conformers I - IV for WHBB, q-AQUA and MB-pol

As anticipated in the Theoretical Details Section, floppy systems like the octamer present several coupled modes, hence QCT frequencies are sometimes swapped between modes for the three PESs.

In the case of Conformer I, WHBB and q-AQUA produce frequencies of 3392 and 3353 cm^{-1} for mode ν_7, and frequencies of $\approx$3440 and $\approx$3430 cm^{-1} for the degenerate modes ν_5 and ν_6. These are swapped for MB-pol, whose frequencies are 3426 cm^{-1} for mode ν_7, and $\approx$3400 cm^{-1} for modes ν_5 and ν_6. Moving to Conformer II, WHBB and MB-pol produce frequencies of 3078 and 3071 cm^{-1} for mode ν_4, and frequencies of $\approx$2990 and $\approx$3000 cm^{-1} for the degenerate modes ν_2 and ν_3. These are swapped for q-AQUA, whose frequencies are 2962 cm^{-1} for mode ν_4, and $\approx$3040 cm^{-1} for modes ν_2 and ν_3. Finally for the two enantiomers, WHBB and MB-pol produce frequencies of 3282 and 3295 cm^{-1} for mode ν_3, and frequencies of 3379 and 3395 cm^{-1} for mode ν_4. These are swapped for q-AQUA, whose frequencies are 3374 cm^{-1} for mode ν_3, and 3289 cm^{-1} for mode ν_4. The same behavior is observed for modes

ν_7 and ν_9. WHBB returns frequencies of 3481 and 3523 cm^{-1}, while q-AQUA and MB-pol give values of ≈3525 and ≈3460 cm^{-1}.

Table S15: Comparison between harmonic (Harm.) and QCT anharmonic frequencies of the experimentally detected modes of Conformers I - IV of $(H_2O)_8$ from the WHBB, q-AQUA and MB-pol PESs. Data are in cm^{-1}. The numeration of the modes refers to the WHBB PES. The equivalent modes for the other PESs are chosen according to their symmetry.

Conf.	Mode	WHBB		q-AQUA		MB-pol	
		Harm.	QCT	Harm.	QCT	Harm.	QCT
I	ν_2	3373	3144	3428	3131	3359	3149
	ν_3	3377	2984	3390	2962	3323	2976
	ν_4		2980		2972		2963
	ν_5	3611	3440	3713	3428	3666	3406
	ν_6		3445		3432		3394
	ν_7	3631	3392	3731	3353	3667	3426
	ν_{11}	3777	3570	3774	3600	3748	3590
	ν_{12}		3595		3568		3575
	ν_{13}	3918	3629	3912	3710	3901	3716
	ν_{14}	3924	3694	3915	3646	3900	3662
	ν_{15}		3687		3647		3662
II	ν_2	3346	2995	3405	3042	3379	3010
	ν_3		2990		3045		2992
	ν_5	3612	3447	3719	3482	3686	3475
	ν_{10}	3749	3532	3764	3545	3746	3524
	ν_{11}		3513		3546		3535
	ν_{12}	3759	3581	3774	3624	3762	3619
III & IV	ν_4	3542	3379	3571	3289	3539	3395
	ν_6	3558	3369	3607	3404	3569	3395
	ν_8	3671	3536	3751	3538	3713	3522
	ν_9	3746	3523	3475	3475	3755	3458
	ν_{13}	3910	3602	3902	3618	3899	3627
	ν_{14}	3930	3722	3914	3695	3903	3695
	ν_{16}	3941	3731	3917	3738	3904	3780

References

(1) Li, G.; Zhang, Y.-Y.; Li, Q.; Wang, C.; Yu, Y.; Zhang, B.; Hu, H.-S.; Zhang, W.; Dai, D.; Wu, G., et al. Infrared spectroscopic study of hydrogen bonding topologies in the smallest ice cube. *Nat. Commun.* **2020**, *11*, 5449.
